# Hospital management and psychosocial risks: challenges and opportunities in the face of regulatory transition

**DOI:** 10.47626/1679-4435-2026-1494

**Published:** 2026-06-15

**Authors:** Fernanda Maria de Miranda, Karine de Oliveira Silva

**Affiliations:** 1 Universidade Pitágoras Unopar Anhanguera, São Carlos, SP, Brazil; Lakehead University, Thunder Bay, ON, Canada; 2 Hospital Universitário da Universidade Federal de São Carlos (HU-UFSCar), São Carlos, SP, Brazil

**Keywords:** mental health, legislation, labor, risk management, hospitals, occupational health.

## Abstract

The update of Regulatory Standard No. 1 issued by the Brazilian Ministry of Labor and Employment represents progress by formally recognizing psychosocial risks as an integral part of occupational risks. This change requires organizations to adopt new practices aimed at preventing illness through more comprehensive assessments of psychosocial risks present in the workplace. This study conducted an integrative review with the objective of analyzing how psychosocial risks have been addressed in the scientific literature related to hospital risk management. Nine articles published between 2019 and 2025 were included. Of these, five directly addressed psychosocial risks, although the hospital environment is recognized as a critical context regarding workers’ mental suffering. Limitations were observed in the use of validated instruments for assessing these risks. The new wording of the standard may act as a catalyst for structural changes in risk management practices, promoting more integrated strategies that consider technical, subjective, and relational aspects of the work environment. It is concluded that, although psychosocial risks are recognized in the theoretical field, their practical incorporation into hospital institutions still occurs incipiently. Strengthening institutional policies and conducting future studies to evaluate the impacts of regulatory changes on the Brazilian hospital reality are recommended.

## INTRODUCTION

Brazilian Regulatory Standard No. 1 (NR-1), issued by the Brazilian Ministry of Labor and Employment (MTE), establishes the requirements for occupational risk management and provides guidance on preventive measures in Occupational Safety and Health (OSH), constituting a regulatory reference for different work settings in Brazil. Recently, NR-1 underwent a significant update resulting from discussions conducted within the Tripartite Study Group, aligned with the guidelines of the International Labour Organization (ILO) and the World Health Organization (WHO) [^1^-^3^]. Among the main changes, the formal recognition of psychosocial risks as an integral part of occupational risks stands out. This is evidenced in the following excerpt from the standard [2:5; free translation]: “occupational risk management must encompass risks arising from physical, chemical, and biological agents, accident risks, and risks related to ergonomic factors, including psychosocial risk factors related to work.”

This regulatory change represents a significant transition in the way Brazilian society understands and addresses psychosocial risks in the workplace. Until then, these risks were often treated as invisible or secondary compared with other occupational hazards that, due to their tangibility, were more easily observed and measured. The formal inclusion of psychosocial risks in NR-1 broadens the debate on traditional risk management models and challenges managers and occupational health teams to adopt more complex approaches, in which interpersonal relationships, work organization, and mental health assume a central role [^4^].

Psychosocial risks were defined by the ILO in 1986 as the result of the interaction between aspects of work, including environment, content, conditions, and interpersonal relationships, and individual worker characteristics, such as abilities, needs, and sociocultural context. This interaction, mediated by individuals’ perceptions and experiences, may represent a risk insofar as it affects health, performance, and job satisfaction [^5^]. The Psychosocial Risk Management Excellence Framework describes examples of these factors, such as continuous exposure to people due to work, high time pressure, night work, lack of control over workload, poor communication, professional stagnation, and lack of family support [^6^]. In this context, the incorporation of psychosocial risks into NR-1 represents not only a technical adaptation but also a paradigm shift in occupational management.

Another relevant change was the integration between NR-1 and NR-17 (Ergonomics), reinforcing psychosocial factors as components of work organization. This integration highlights that the identification and assessment of psychosocial risks should consider the actual activities performed by workers rather than only formally prescribed tasks. The assessment may occur qualitatively, based on technical observation and the experience of OSH professionals, supported by validated instruments when appropriately applied. This process should not be mistaken forindividual clinical assessments, as its focus lies on working conditions rather than on workers’ health status [^1^].

The hospital environment, especially in the context of nursing, stands out as one of the most critical settings regarding work-related illness and mental suffering [^7^]. Scientific literature consistently points to the presence of psychosocial risks in this sector [^8^,^9^], a reality intensified after the COVID-19 pandemic. Although several mental health promotion strategies have been implemented, there is a predominance of approaches centered on the individual, with limited emphasis on collective and organizational aspects of work, as well as reduced employer participation in the proposed interventions [^10^].

Updating NR-1 is an important advance by explicitly incorporating psychosocial risks as an object of institutional management. This change is aligned with international guidelines aimed at developing organizational strategies to promote mental health at work [^11^] and proposes a new perspective for institutional and preventive approaches. It is worth noting that previous versions of the standard had already introduced important advances, such as the incorporation of measures to prevent and combat sexual harassment and other forms of workplace violence, as established by Ordinance No. 4,219 of the Brazilian Ministry of Labor and Social Security [^12^]. However, the new wording broadens the understanding of organizational co-responsibility in workers’ mental illness.

Because of the recent update of NR-1, reflecting on the possible impacts of its implementation in the hospital context becomes relevant, particularly because psychosocial risks manifest in complex ways and remain insufficiently systematized within the field of risk management. This study has social and academic relevance by analyzing how these risks have been addressed in scientific literature published prior to the regulatory change, seeking to provide support for future institutional adaptations and for strengthening the theoretical framework related to occupational risk management in hospitals. Therefore, this study aims to analyze how psychosocial risks have been incorporated into scientific literature on hospital risk management.

## METHODS

An integrative review methodological design was adopted, encompassing the stages of problem identification, literature search and screening, data evaluation and analysis, and review presentation [^13^]. The results were described according to the recommendations of the Preferred Reporting Items for Systematic Reviews and Meta-Analyses (PRISMA), with the aim of ensuring greater quality and transparency in the review process [^14^]. Items from the checklist considered not applicable to the integrative review design were disregarded since PRISMA was originally developed for systematic reviews focused on health interventions.

The protocol for integrative review was registered at the Center for Open Science through OSF Registries in September 2024 and updated in January 2026 to expand and update the search strategy. The full protocol is available through the DOI 10.17605/OSF.IO/MJPV4.

The study was conducted based on the following guiding question: “How have psychosocial risks been included in scientific literature on occupational risk management for health professionals in the hospital context?” The development of the question was based on the PCC mnemonic, in which population (P) corresponded to health professionals; concept (C), to psychosocial risks; and context (C), to hospital risk management.

The information sources included were BDENF and LILACS, via the Virtual Health Library; MEDLINE, via PubMed; Web of Science; Embase; CINAHL; and SciELO.

The search strategy was developed based on the controlled descriptors from the Health Sciences Descriptors and Medical Subject Headings: “Risk Management,” “Occupational Health,” and “Hospitals,” as well as their respective synonyms, combined using Boolean operators (Chart 1).

**Chart 1:** Search strategies by database

Databases	Search strings
BDENF/LILACS	(gestão de riscos OR notificação de incidentes hospitalares OR notificações de incidentes hospitalares OR notificação de riscos hospitalares OR notificações de riscos hospitalares OR notificação de incidentes OR notificação de incidentes em hospitais OR notificações de incidentes OR gestão de risco OR gestão de riscos OR notificação voluntária de eventos de segurança do paciente) AND (hospitais OR hospital) AND (saúde ocupacional OR saúde do trabalhador OR saúde dos trabalhadores OR saúde industrial OR higiene industrial OR higiene ocupacional OR segurança ocupacional OR segurança do trabalho)
SciELO	(riscos ocupacionais OR risco ocupacional OR riscos psicossociais OR gerenciamento de riscos OR gerenciamento de risco) AND (hospital OR hospitais)
MEDLINE	(psychosocial risk OR psychosocial risks OR psychosocial risks factors at work) AND (risk management OR hospital incident reporting OR hospital incident reportings OR hospital risk reporting OR hospital risk reportings OR incident reporting OR incident reporting, hospital OR incident reportings OR incident reportings, hospital OR management, risk OR management, risks OR reporting, hospital incident OR reporting, hospital risk OR reporting, incident OR reportings, hospital incident OR reportings, hospital risk OR reportings, incident OR risk reporting, hospital OR risk reportings, hospital OR risks management OR voluntary patient safety event reporting) AND (hospitals OR hospital) AND (occupational health OR employee health OR health, employee OR health, industrial OR health, occupational OR hygiene, industrial OR industrial health OR industrial hygiene OR occupational safety OR safety, occupational)
Web of Science/Embase/CINAHL	(risk management OR hospital incident reporting OR hospital incident reportings OR hospital risk reporting OR hospital risk reportings OR incident reporting OR incident reporting, hospital OR incident reportings OR incident reportings, hospital OR management, risk OR management, risks OR reporting, hospital incident OR reporting, hospital risk OR reporting, incident OR reportings, hospital incident OR reportings, hospital risk OR reportings, incident OR risk reporting, hospital OR risk reportings, hospital OR risks management OR voluntary patient safety event reporting) AND (hospitals OR hospital) AND (occupational health OR employee health OR health, employee OR health, industrial OR health, occupational OR hygiene, industrial OR industrial health OR industrial hygiene OR occupational safety OR safety, occupational) AND (Brazil OR Brasil OR brasil^*^ OR brasil$ OR Brazil^*^ OR Brazi$)

Inclusion criteria comprised Brazilian articles published in Portuguese, English, or Spanish between 2019 and 2025. The geographic restriction was defined due to the national applicability of NR-1. The time frame was established to identify more recent approaches to risk management, considering the updates promoted by Ordinance No. 915 of the Special Secretariat for Social Security and Labor [^15^] and Ordinance No. 1,419 of MTE [^2^,^3^].

Dissertations, theses, literature reviews and studies that mentioned risk management but did not address its planning, implementation, evaluation, or reflection were excluded.

Article selection was performed using the Rayyan app [^16^] in two stages: initially, the titles and abstracts of publications identified in the search were screened; subsequently, the preselected studies were evaluated through full-text review, applying the inclusion and exclusion criteria.

Screening was conducted by a single researcher. Although this strategy represents a methodological limitation, its adoption was justified by the practical feasibility of the research and the impossibility of conducting a double-blind process, considering that the study originated from an individual monograph developed within the context of a lato sensu specialization course.

**Figure 1:**
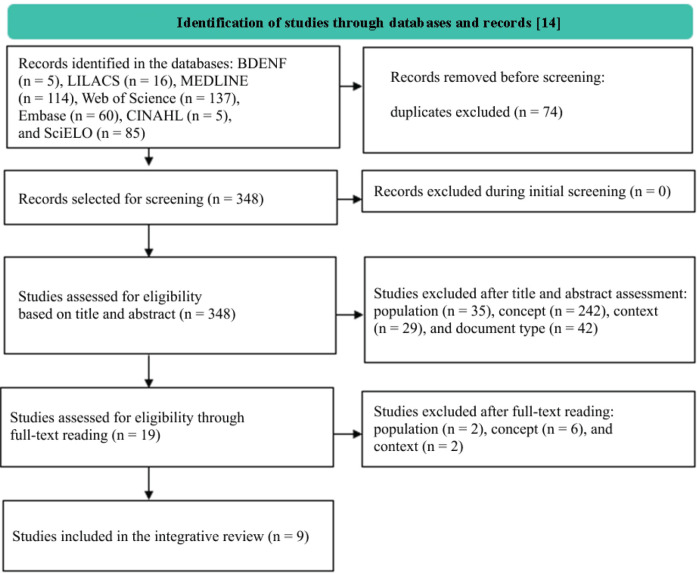
Article selection stages.

Searches were conducted on September 17, 2024, and updated on January 15, 2025, initially resulting in 422 publications. After the screening and analysis stages, the final sample consisted of nine articles (Figure 1).

For data evaluation, the articles included in the sample were classified according to level of evidence (LoE) and underwent critical appraisal (CA) using the Johns Hopkins Nursing Evidence-Based Practice Research Evidence Appraisal Tool [^17^], an instrument suitable for studies with quantitative, qualitative, and mixed-methods designs.

LoE was categorized as follows: level I, corresponding to randomized clinical studies; level II, to quasi-experimental studies; and level III, to nonexperimental studies. CA was classified into three categories: high quality (A), good quality (B), and low quality (C).

The analysis of the included articles was conducted descriptively with the aid of a matrix developed in Excel. Data extracted included study characteristics, such as year of publication, authorship, objective, study type, target population, and methodological limitations, as well as the psychosocial factors identified and the respective risk management strategies described.

Data were organized based on the theoretical-conceptual framework of psychosocial risks [^5^] and the regulatory guidelines of the updated NR-1 [^1^-^3^].

## RESULTS AND DISCUSSION

All studies included in the sample were classified as nonexperimental (LoE III) and assessed as having good methodological quality (classification B), according to the LoE and CA appraisal tool used [^17^]. This homogeneous pattern highlights the predominance of well-conducted observational studies in the scientific literature on psychosocial risks in the hospital context.

In order to facilitate the presentation of the findings, studies were organized into two charts. Chart 2 includes studies with quantitative, mixed-methods, or methodological approaches, whereas Chart 3 includes studies with qualitative approaches. This organization aims to highlight the methodological diversity and the different approaches adopted by the studies in identifying, characterizing, and analyzing occupational risks.

**Chart 2:** Characterization of quantitative and mixed-methods studies (n = 6)

Year [Article Number]	Objective	Method	Occupational risks	Results/Conclusions
2024 [^18^]	To analyze exposure to ergonomic risks and the occurrence of musculoskeletal pain among hospital cleaning service workers	Mixed-methods study, including observation, photographic records, questionnaires, and convergence groups (n = 149)	Ergonomic	Exposure to ergonomic risks showed a multifactorial nature, including inadequate postures, repetitive movements, prolonged standing, and the use of equipment not adapted to workers’ psychophysiological needs.
2021 [^19^]	To perform the translation and cross-cultural adaptation of the instrument Risk assessment and management of exposure of health care workers in the context of COVID-19	Methodological study of translation and cross-cultural adaptation of a measurement instrument (n = 35)	Biological; accidents	The instrument was validated for Brazilian Portuguese, showing good applicability and satisfactory reliability.
2021 [^20^]	To analyze the level of psychosocial risks among workers in the central sterile supply department of a large hospital in Rondônia	Quantitative, descriptive, and cross-sectional study (n = 35)	Psychosocial	The dimension related to justice and respect showed high psychosocial risk, whereas four other dimensions showed moderate risk.
2021 [^21^]	To investigate the characteristics of work organization in the central sterile supply department and analyze nursing workers’ exposure to psychosocial risks	Mixed-methods study (n = 36; 19)	Psychosocial	A moderate overall psychosocial risk was identified. Reports of pleasure at work were associated with professional recognition, teamwork, and indirect patient care. Suffering was related to insufficient staffing and supplies, maintenance failures, inadequate physical space, communication problems, sick leave, and institutional devaluation.
2020 [^22^]	To identify the knowledge of nursing workers in the emergency department of a university hospital regarding the occupational risks to which they are exposed	Qualitative, descriptive, and exploratory study (n = 137)	Physical; chemical; biological; ergonomic	Professionals recognized biological risks as the main occupational risks present in health care practice, followed by physical, accident-related, and chemical risks.
2019 [^23^]	To analyze nonclinical risks in a central sterile supply department	Observational, analytical, and longitudinal study	Ergonomic; burns; electric shock; fire; biological; water quality	A higher frequency of factors classified as moderate risk was observed.

**Chart 3:** Characterization of qualitative studies (n = 3)

Year [Article Number]	Objective	Method	Occupational risks	Results/Conclusions
2020 [^24^]	To evaluate the occupational factors affecting the health of nursing professionals in the hospital context	Qualitative, descriptive, and exploratory study (n = 22)	Ergonomic; accident-related; chemical; physical; biological; psychosocial	The existence of occupational risk management actions was observed; however, a need for greater integration of these practices into the hospital routine was identified. As a product of the study, a biological risk management protocol was developed.
2020 [^25^]	To investigate the impacts of work organization on the daily routine of nursing care in the surgical center of a general hospital	Qualitative, descriptive-exploratory study with an anthropological approach (n = 4)	Physical; chemical; biological; mechanical; ergonomic; psychosocial	Exposure to psychosocial risks proved to be central to work dynamics, leading nurses to develop informal, individual, and collective risk and safety management strategies, such as self-management and the implicit use of the right to refuse, in the absence of formal institutional mechanisms.
2019 [^26^]	To characterize the presence of work-related psychosocial risks among nurses in a psychiatric hospital and the strategies used to manage these risks	Qualitative, descriptive, and exploratory study (n = 25)	Psychosocial	The following work-related psychosocial risks were identified: insufficient academic training; deficiencies in equipment preparation and maintenance; weakened interpersonal relationships; shortage of human resources; lack of professional training; and conflicts between family and work demands.

Although the population investigated in the analyzed studies consisted of health care workers [^20^-^26^], most studies focused exclusively on the risks faced by the nursing staff, with hospital cleaning workers [^18^] being the only other professional group specifically analyzed. The studies were conducted in both general hospital settings [^19^,^24^] and specialized scenarios, such as psychiatry [^26^], emergency care [^22^], surgical centers [^25^], and central sterile supply departments [^20^,^21^,^23^]. Although the hospital environment is widely recognized as a high-risk context for mental health disorders, only five articles (55.6%) explicitly addressed psychosocial risks.

The time frame adopted (2019-2025) preceded the implementation of the new wording of NR-1, scheduled for 2026. Thus, the results reflect how psychosocial risks had already been considered in scientific literature, even without explicit regulatory provisions. The recent update of NR-1 institutionalizes this recognition, providing regulatory support for a more systematic and integrated approach to these risks. Although the previous wording of the standard already allowed situations of workplace violence to be framed as hazards related to work organization and working conditions, there was no explicit recognition, specific guidelines, or requirement for systematic assessment of these events, which may have contributed to the still incipient approach identified in the analyzed studies. The regulatory update is expected to strengthen both scientific production and institutional practices aimed at managing psychosocial risks.

The factors identified in the included studies are aligned with the definitions proposed by ILO [^5^]. Among the main psychosocial risks described were psychological overload resulting from excessive work and accumulation of tasks [^24^]; work-related, cognitive, and emotional demands [^20^,^26^]; social relationships and leadership, including insufficient support from colleagues and supervisors as well as interpersonal conflicts [^20^,^26^]; work organization and task content [^20^,^21^]; work-individual interface, with repercussions on physical and mental health [^24^,^26^]; and aspects related to justice and respect in interpersonal relationships [^20^].

Other elements associated with occupational stress included the complexity, invisibility, and high responsibility of the activities performed, use of inadequate equipment, communication failures, and administrative overload [^21^,^25^,^26^]. On the other hand, emotional involvement and identification with patient care were recognized as positive factors for professional engagement and motivation [^21^,^25^].

Considering the interactional nature of psychosocial risks, which simultaneously involves characteristics of the work environment and individual worker aspects, it is essential that the management of these risks encompasses both the individual and collective dimensions of work [^10^]. Thus, effective management of psychosocial risks should be based on strategies that consider not only objective working conditions but also workers’ subjectivity, including interpersonal relationships, expectations, and life experiences [^4^].

In this context, ergonomics emerges as one of the relevant approaches for assessing psychosocial risks, especially given the integration between NR-1 and NR-17, which recognizes these risks as components of work organization. Through analysis of actual work activity and qualified listening to workers, ergonomics makes it possible to capture subjective and contextual aspects that are often not considered in exclusively quantitative assessments. This approach favors the identification of specific and dynamic psychosocial factors, guiding interventions that are better suited to workers’ concrete realities and contributing to the promotion of mental health and to the structured and preventive improvement of working conditions [^27^].

Regarding the assessment of psychosocial risks, one of the included studies [^20^] used the Brazilian version of the Copenhagen Psychosocial Questionnaire [^28^], whereas another [^21^] employed the *Escala de Organização Prescrita do Trabalho*. Using questionnaires for assessing psychosocial risks appears promising, as it allows the use of well-defined, comprehensive, and systematized constructs, promoting a more accurate understanding of the work context. However, the findings reveal a substantial gap between the theoretical recognition of psychosocial risks and their practical incorporation into occupational assessment and management processes. Although the relevance of these risks is widely recognized in the studies, the adoption of systematic approaches associated with qualitative techniques remains limited, and the use of validated instruments is still infrequent. This methodological limitation reinforces the need for continuous training of occupational health professionals and for broader use of specific tools to assess psychosocial risks [^1^].

The predominant logic in psychosocial assessment, in turn, tends to fragment risks into quantifiable categories, often disregarding broader historical, social, and organizational determinants that influence working conditions. This model may favor the individualization of mental illness, holding workers responsible for adapting to the work environment instead of promoting structural changes in working conditions [^29^]. The qualitative and subjective nature of psychosocial factors therefore requires broader approaches capable of integrating quantitative and qualitative dimensions of work, including workers’ individual and collective experiences [^4^].

Furthermore, the recent update of NR-1 encourages revisions in other regulations and promotes a more integrated and multidimensional approach to occupational risks, particularly in the hospital sector. NR-9, for example, was used to categorize occupational risks in one of the analyzed studies. In this context, a dichotomy can be observed between the theoretical recognition of psychosocial risks, expressed in the introductory excerpt of the study [22; free translation], and their absence in the effective analysis of occupational risks, considering the framework adopted by the authors based on NR-9: “…chemical, physical, biological, psychological, ergonomic, and organizational risks, especially when acting in environments without adequate working conditions, highly unhealthy, and lacking the availability of Personal Protective Equipment.”

## FINAL CONSIDERATIONS

The update of NR-1, by explicitly incorporating psychosocial risks, represents a significant advance in the institutional recognition of the impacts of work on workers’ mental health. Through this study, it was possible to observe that psychosocial risks have still been insufficiently incorporatedin the scientific literature related to hospital risk management, although all nonexperimental studies analyzed demonstrated good methodological quality.

The current historical context, however, requires a deeper reflection on the risk management practices adopted in health care institutions. The regulatory change represents an opportunity to rethink existing management models, encouraging more complex and integrated approaches capable of encompassing not only the technical aspects of work but also interpersonal relationships, organizational culture, and the social dynamics that permeate the work environment.

In the hospital context, this regulatory transition may act as a catalyst for transformative changes, both in the management of occupational risks and in the promotion of mental health and well-being among health professionals. Future studies are recommended to conduct comparative analyses across different institutional settings over time, using the findings of this review as a reference. In this way, it will be possible to contribute to the improvement of occupational health regulations and public policies, as well as to document in the scientific literature the possible impacts of the regulatory transition on hospital risk management.

## Data Availability

Upon publication, the data will be made available by the authors upon request
